# Cannabis consumption and prosociality

**DOI:** 10.1038/s41598-022-12202-8

**Published:** 2022-05-19

**Authors:** Jacob Miguel Vigil, Sarah S. Stith, Tiphanie Chanel

**Affiliations:** 1 Department of Psychology, University of New Mexico, Albuquerque, USA; 2 Department of Economics, University of New Mexico, Albuquerque, USA

**Keywords:** Plant sciences, Psychology, Medical research

## Abstract

The existing literature largely focuses on health risks and other pharmacodynamics of using cannabis, with fewer investigations of other normative psychological effects from consumption among otherwise healthy people. We measured several basic constructs of social psychology corresponding to the concept of prosociality among 146 healthy young adults between 18 and 25 years (*M* = 18.9, *SD* = 1.4) with varying detectable levels of tetrahydrocannabinol (THC) in their urine, controlling for participant’s sex, age, ethnicity, and childhood socio-economic status. Compared to THC-free individuals, cannabis users scored higher than non-users on validated measures of Prosocial Behaviors (*d* = .34, *p* = .04), the Empathy Quotient (*d* = .36, *p* < .01), Moral Harmlessness (*d* = .76, *p* < .01) and Moral Fairness (*d* = .49, *p* < .01), but exhibited a lower sense of Ingroup Loyalty (*d* = .33, *p* = .04). Relative to THC-free, same-sex individuals, female cannabis users scored significantly higher on measurements of Aggression (*d*s = .65 and .57, *p*s < .05) and male users scored higher on the Agreeableness dimension of personality (*d* = .91, *p* < .01).. Linear associations were found between the recency of last cannabis usage and the Prosocial Behaviors, Empathy Quotient, Moral Harmlessness, Moral Fairness and Agreeableness personality scores (*r*s from − .24 to .38, *p*s < .05). The findings suggest cannabis usage is associated with an increased sense of prosociality and prioritization of humanitarian behaviors that declines with time following cannabis consumption. Further research should focus on heterogeneity in the effects of cannabis consumption across users.

## Introduction

Due in part to its nearly century-long designation as an illicit substance by the federal government of the United States, the scientific community has mostly focused on the pharmacodynamics and health risks of consuming the *Cannabis* plant, with much fewer investigations of its potential effects on other basic elements of normative psychological functioning. For example, “prosociality” refers to the intentional act of advancing the well-being of other people^1,2^. Prosocial behaviors, such as displays of empathy, providing assistance to others, and engaging in community service, not only enhance the individual’s social status, but also promote distinct health advantages^1,3^. Individuals that voluntarily engage in higher rates of prosociality benefit from greater physical health, lower disease rates, higher quality of life, and longer average lifespans^4–8^. Psychologically, prosociality induces feelings of happiness, which in turn increase the motivation to engage in further acts of prosocialty^9–12^, thereby creating a positive behavioral health loop for the actor^13^. However, because prosociality also entails a direct benefit to a target, which can often result in tertiary beneficence beyond the initial actor/target, prosociality can be considered not only essential to, but also an accurate metric of a society’s overall cohesiveness and vitality^1^.

While few (if any) documented studies have attempted to measure the association between cannabis use and prosocial behaviors, numerous investigations have measured how using cannabis may affect antisocial behaviors. For example, one study among people with a criminal history of aggression found that cannabis use predicted violence and that violence in turn predicted cannabis use, leading the authors to conclude: “continued cannabis use remained the strongest predictor for subsequent violent conviction,” even more so than alcohol use^14^. Among men arrested for domestic violence, cannabis consumption has also been shown to predict subsequent perpetration of intimate partner violence^15^. In community samples, self-reported cannabis consumption has been found to temporally correlate with both physical partner violence^16^ and with a greater likelihood of partner conflict and verbal aggression^17^. Cannabis withdrawal symptoms can also include increased irritability and aggression^18^, and withdrawal has been estimated to result in a 60% higher odds of past year relationship aggression^19^. Other researchers have even so much as estimated that “a 10% increase in cannabis (use) frequency is associated with a 0.4% increase in the frequency of violent behavior”^20^.

Contrasting with those findings, other researchers have concluded that prenatal and perinatal cannabis exposure has a minimal (if any) effect on aggressive behavior, and the links between postnatal cannabis use and risk of psychosocial problems is merely correlational, with many possible alternative explanations, including selection bias, measurement imprecision, preexisting or predisposed psychological problems, and reverse direction of causality. There also exists the possibility that cannabis may often be consumed to self-medicate feelings of anger and aggression^21–23^. Moreover, landmark animal studies^24^ and recent experimental research in humans^25^ have shown that, unlike alcohol^26^, acute cannabis intoxication results in a decrease in aggressive behaviors and subjective feelings of aggression. Large observational recordings of real-time cannabis usage sessions also show that the majority of users experience anxiolytic effects and reductions in feelings of irritability/agitation, stress, and mood swings following consumption of commercially available cannabis^27,28^. Among clinical samples, such as people with a diagnosis of schizophrenia, cannabis use is associated with lower levels of psychotic symptoms and increased social behaviors^29–31^. The plant’s ability to stimulate the CB1 and CB2 receptors in the central nervous system and modulate the expression of other neurotransmitters such as serotonin is similar to conventional antipsychotic pharmaceutical medications^32,33^, thus prompting many researchers to now suggest cannabis as a potential treatment option for a wide spectrum of health conditions that affect mood^34–37^. Still, emotion processing studies show mixed results, with some researchers observing differences in brain activity (e.g., EEG, FMRI) when attending to, or in the accuracy of identifying discrete emotional stimuli^38–40^, but other researchers not observing statistically significant effects of cannabis exposure^41^.

The current study extends the clinical and psychological literatures by measuring the associations between cannabis consumption and normative social psychological functioning in otherwise healthy young adults. Objective and subjective measurements of recent cannabis use are compared to self-reported measurements of trait aggression, prosocial behaviors, empathy, trustfulness of peers, personality characteristics, moral reasoning foundations, and visual facial threat perceptions among a convenience sample of university students. While this sample is likely disparate for many circumstances (socioeconomic status [SES], background of criminality and exposure to violence, etc.) from a non-university community sample, the current sample is ideal for measuring relative differences in the associations between cannabis exposure and psychological functioning among a healthy, young adult population with relatively homogeneous life circumstances. With widespread medical authorizations and increasing recreational allowance of cannabis use throughout the U.S., it is vital to understand how such transitions may affect normative individual-level patterns of behavior and their potential societal impact.

## Methods

### Sample

The research protocol was approved by the University of New Mexico’s Institutional Review Board. The study was performed in accordance with approved guidelines and regulations, and written informed consent was provided by all participants. The sample consisted of 146 undergraduate students, who enrolled in a study titled “Cannabis Consumption and Social Psychological Functioning in Healthy Young Adults.” These students were recruited from the general research pool of undergraduate, psychology students, who exchange their participation in local research for course credits as part of the general curriculum. In order to increase the generalizability of our sample, the only exclusionary criteria consisted of an age restriction (younger than 18 years or older than 25 years) and the absence of any chronic underlying health conditions. In addition to course credits, participants were provided a small monetary reimbursement ($15.00) for their participation, and no attrition occurred in the study. Importantly, enrollment and study procedures occurred prior to any COVID-19-related safety mandates, travel restrictions, or wider mental and physical health detriments caused by COVID-19 and related policies. In total, 46% (*n* = 67) of participants reported current or recent cannabis use. The correlation between participants’ self-reported cannabis use and their urine analyses was nearly perfect (*r* = .96), with 1 participant reporting to have recently used cannabis, but showing a negative urine analysis, and 2 participants denying recent cannabis use but showing a positive urine analysis. For these three participants, user category was based off their urine analyses, resulting in a total of 68 cannabis users (mean age = 19.0 yrs., *SD* = 1.6, 63% females) and 78 non-users (mean age = 18.9 yrs., *SD* = 1.3, 62% females). As shown in Table 1, Chi-square and independent samples *t*-tests showed the proportion of females, average age, ethnic identity, parents’ education levels, and childhood household income of the participants were not significantly different across the user groups (*p*s > .05).

**Table 1:** Descriptive characteristics of non-users and cannabis users.

Subject characteristics	Non-users (*n* = 78)	Users (*n* = 68)	Effect statistic
% Females	63%	62%	0.02 (Chi-square)
Age	18.88 (1.27)	18.97 (1.61)	− .36 (*t*-test)
Ethnicity			.14 (Chi-square)
Non-hispanic white	35%	32%	–
Hispanic	51%	54%	–
Other	14%	13%	–
Socio-economic status			
Mother’s education level	4.28 (2.51)	4.34 (2.41)	− .14 (*t*-test)
Father’s education level	4.00 (2.49)	3.99 (2.37)	.04 (*t*-test)
Childhood household income	3.82 (2.54)	3.10 (2.21)	1.80 (*t*-test)

All group comparisons yielded non-significant differences, *p*s > .05.

### Study procedures

Participants first met with the investigators to consent to the study, provide a urine sample, and complete a battery of psychological tests (see Supplemental Materials), along with assessments (e.g., dietary intake) and physical measurements (e.g., body-fat analyses) unrelated to the current treatment.

#### Urine analyses

After providing consent, participants provided a urine sample, and Narcocheck Extended version PreDosage Cannabis (THC) test kits^42^ were used to determine cannabis exposure. The disposable kits provide the results in 5 min and are able to detect the presence of several cannabinoids, including tetrahydrocannabinol (THC) levels at a threshold of 18 ng/ml.

#### Materials

Trait aggression was measured with the *Aggression Questionnaire*^43^.The 12-item scale consists of four major components of aggression (physical aggression, verbal aggression, anger and hostility), and the items were scored on a 5-point scale (from *extremely uncharacteristic of m*e to *extremely characteristic of me* [overall *α* = .70]). Perceived trustworthiness of peers was measured with a modified version of the *Trust Scale*^44^, which is designed to measure the perceived trustworthiness of other people. The instrument consists of 3 subscales (predictability, dependability, and faith in others) and includes a total of 18 items that were modified to refer to the perceived trustworthiness of one’s “peers”, and scored on a 7-point scale (from *strongly disagree* to *strongly agree* [overall *α* = .86]). The *Prosocial Behaviors Scale*^45^ was used to measure individual variability in prosocial behaviors and attributes (e.g., “I try to help others,” “I spend time with those friends who feel lonely”). The 16 items were scored on a 5-point scale (from *never* to *always*) and demonstrated strong reliability [*α* = .87]). The *Empathy Quotient*^46^ was used to measure the ability to detect, and sensitivity to respond to the feelings of others (e.g., “I really enjoy caring for other people,” “I am good at predicting what someone will do”). The 22 items were scored on a 7-point scale (from *strongly disagree* to *strongly agree* [overall *α* = .80]). Personality characteristics were measured with the *Ten-Item Personality Inventory*^47^. The items measure the “Big 5” dimensions of personality (extraversion, agreeableness, conscientiousness, emotional stability, and openness to experiences) on a 7-point scale (from *disagree strongly* to *agree strongly* [overall *α* = .71]). Moral reasoning was measured with the *Moral Foundations Questionnaire*^48^. The scale measures the reliance on, and endorsement of five psychological foundations of morality based on moral foundations theory. Each of the two parts of the scale contained four questions related to each foundation: (1) harm/care, (2) fairness/reciprocity (including issues of rights), (3) ingroup loyalty, (4) authority/respect, and (5) purity/sanctity. The items are scored on a 5-point scale (from *not at all relevant* to *extremely relevant* [part 1] and from *strongly disagree* to *strongly agree* [part 2] [overall *α* = .79]). Finally, visual facial threat perceptions were measured using facial stimuli created with FaceGen software^49^. Six ambiguous facial stimuli were created by simultaneously setting the facial expression parameters of the software to the maximum levels for two discrete emotions, for every combination of emotions, from a total of four distinct emotions: sadness, joy, fear, and anger. Under each sketch, participants were instructed to identify the facial expression as displaying either: *anger* (A)*, joy* (J)*, fear* (F)*,* or *sadness* (S). Participants’ threat perception responses were coded according to whether the reported emotion facilitates the affiliative (joy or sadness coded 0) or avoidant (anger or fear coded 1) dimensions of human emotionality^3^. Latency since last cannabis consumption was measured on a 9-point scale from “within the past few hours” to “over a year ago”.

### Statistical analysis

Multivariate regressions were used to examine the separate and interactive effects of participants’ user status and biological sex on the construct measurements with age, childhood income level, and ethnic identity (coded 0 = Non-White, 1 = White) included as covariates. Independent-sample *t*-tests were used to compare raw group mean differences, and effect sizes pertaining to group comparisons were estimated with Cohen’s *d* (mean difference/mean standard deviation^50^) and partial correlations. Analyses were conducted using SPSS V.21.

## Results

Table 2 shows the mean values for the psychological constructs across the two user groups.

**Table 2:** Raw mean construct scores for nonusers and cannabis users.

Construct	Nonusers	Users	*t*-test	Cohen’s *d*
Aggression (overall score)	33.03 (6.96)	35.49 (7.01)	− 2.08*	.35
Physical	7.18 (2.85)	7.90 (3.10)	− 1.45	.24
Verbal	9.77 (2.48)	10.40 (2.38)	− 1.56	.26
Anger	7.61 (1.73)	7.91 (2.02)	− .95	.16
Hostility	8.39 (2.95)	9.28 (2.65)	− 1.90	.32
Peer trust (overall score)	14.23 (15.46)	12.29 (14.98)	.76	.13
Predictability	5.19 (4.78)	4.25 (4.03)	1.26	.21
Dependability	5.45 (7.39)	4.90 (6.79)	.47	.08
Faith	3.39 (6.36)	3.15 (6.92)	.22	.04
Prosocial behaviors	14.92 (7.81)	17.63 (8.10)	− 2.00*	.34
Empathy quotient	1.13 (.68)	1.34 (.60)	− 1.94	.33
Big 5 personality (overall score)	7.55 (8.68)	7.35 (8.22)	.14	.02
Extraversion	− 00.13 (3.37)	00.08 (3.48)	− .36	.06
Agreeableness	1.49 (2.13)	1.91 (1.75)	− 1.30	.22
Conscientiousness	2.88 (2.45)	2.36 (2.59)	1.25	.21
Emotional stability	.73 (3.02)	.12 (2.97)	1.22	.20
Openness	2.65 (2.54)	2.84 (2.16)	− .47	.08
Moral foundations (overall score)	62.44 (10.94)	63.05 (11.60)	− .32	.05
Harmlessness	14.74 (2.41)	16.60 (2.46)	− 4.58***	.76
Fairness	15.08 (2.66)	16.37 (2.54)	− 2.94***	.50
Ingroup loyalty	11.00 (3.73)	9.81 (3.55)	1.96*	.33
Respect for authority	11.09 (3.62)	9.99 (4.14)	1.72	.28
Purity	10.99 (4.26)	10.38 (4.18)	.86	.14
Facial threat perceptions	3.27 (.79)	3.15 (.75)	.88	.16

*p < .05, **p < .01, ***p < .001.

In order to examine whether cannabis user status interacts with the participants’ biological sex in their association with the psychological outcome variables, User Group, Sex, and the User Group x Sex interaction terms were entered as predictor variables along with the covariates: age, childhood household income, and ethnicity. A significant User Group x Sex interaction term emerged for the overall Aggression score, *B* = 4.62, *t* = 1.98, *p* = .05; the Physical Aggression subscore, *B* = 2.42, *t* = 2.49, *p* = .014; and the Agreeableness personality characteristic, *B* = − 2.23, *t* = − 3.43, *p* = .001. The interaction term was not significant for the remainder of the outcome variables (*p*s > .05).

**Figure 1:**
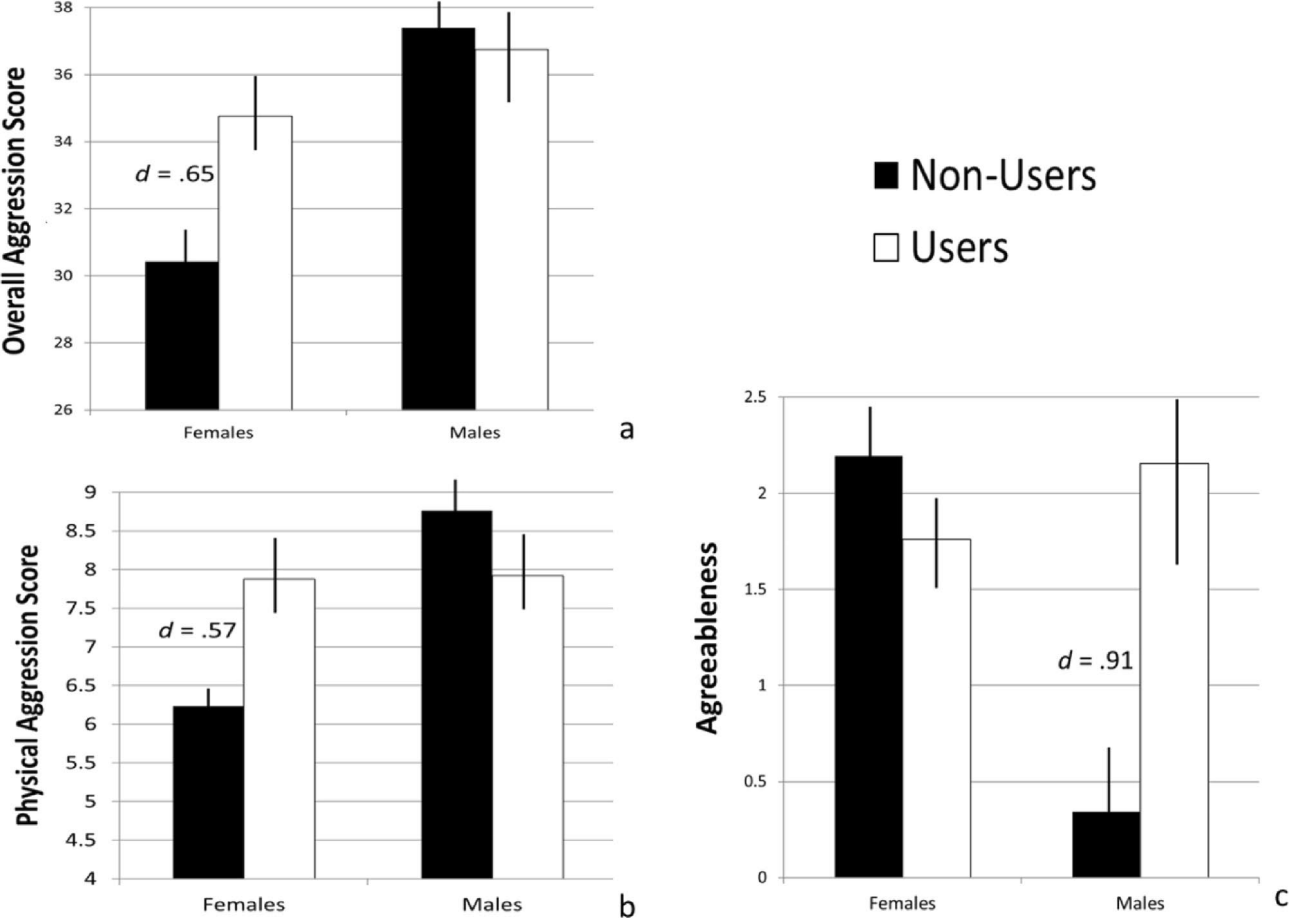
Group differences in overall aggression, physical aggression, and agreeableness construct scores. Note. The total (overall) aggression scores ranged from 17 to 50, physical aggression scores 3 to 14, and agreeableness scores − 5 to 6.

Follow-up analyses examining the effect of User Group on the overall aggression and the physical aggression scores separately for women and men (including the covariates: age, income, and ethnicity) showed a significant effect of User Group among women for the overall Aggression score, *B* = 3.81, *t* = 2.58, *p* = .01; and the Physical Aggression subscore, *B* = 1.47, *t* = 2.35, *p* = .02. The effect of User Group was not significant for men (*p*s > .20). These effects are illustrated in Fig. 1a and b, which show female cannabis users self-report significantly higher levels of Aggression (*d*s = .65 and .57) than non-using women, with no such effect observable among men. In contrast, a similar follow-up analysis for the Agreeableness personality characteristic showed a significant effect of User Group for men only, *B* = 1.75, *t* = 3.17, *p* < .01, while the effect of User Group was not significant for women (*p* = .19). As shown in Fig. 1c, male cannabis users show significantly higher levels of personality characteristics associated with Agreeableness (*d* = .91) as compared to non-using men, while no such effect was detectable among women.

**Figure 2:**
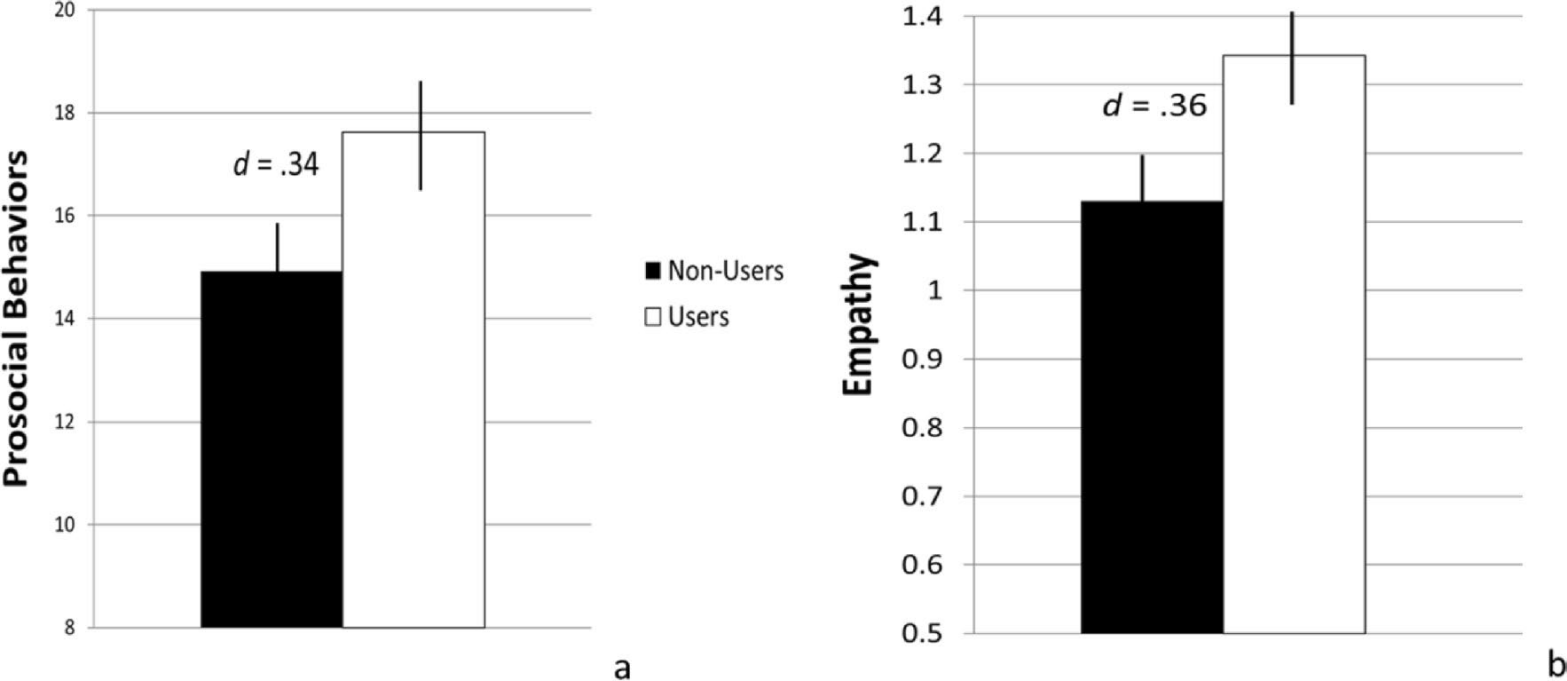
Group differences in prosocial behaviors and empathy quotient construct scores. Note. The prosocial behaviors scores ranged from − 8 to 32 and empathy scores − .15 to 2.60.

**Figure 3:**
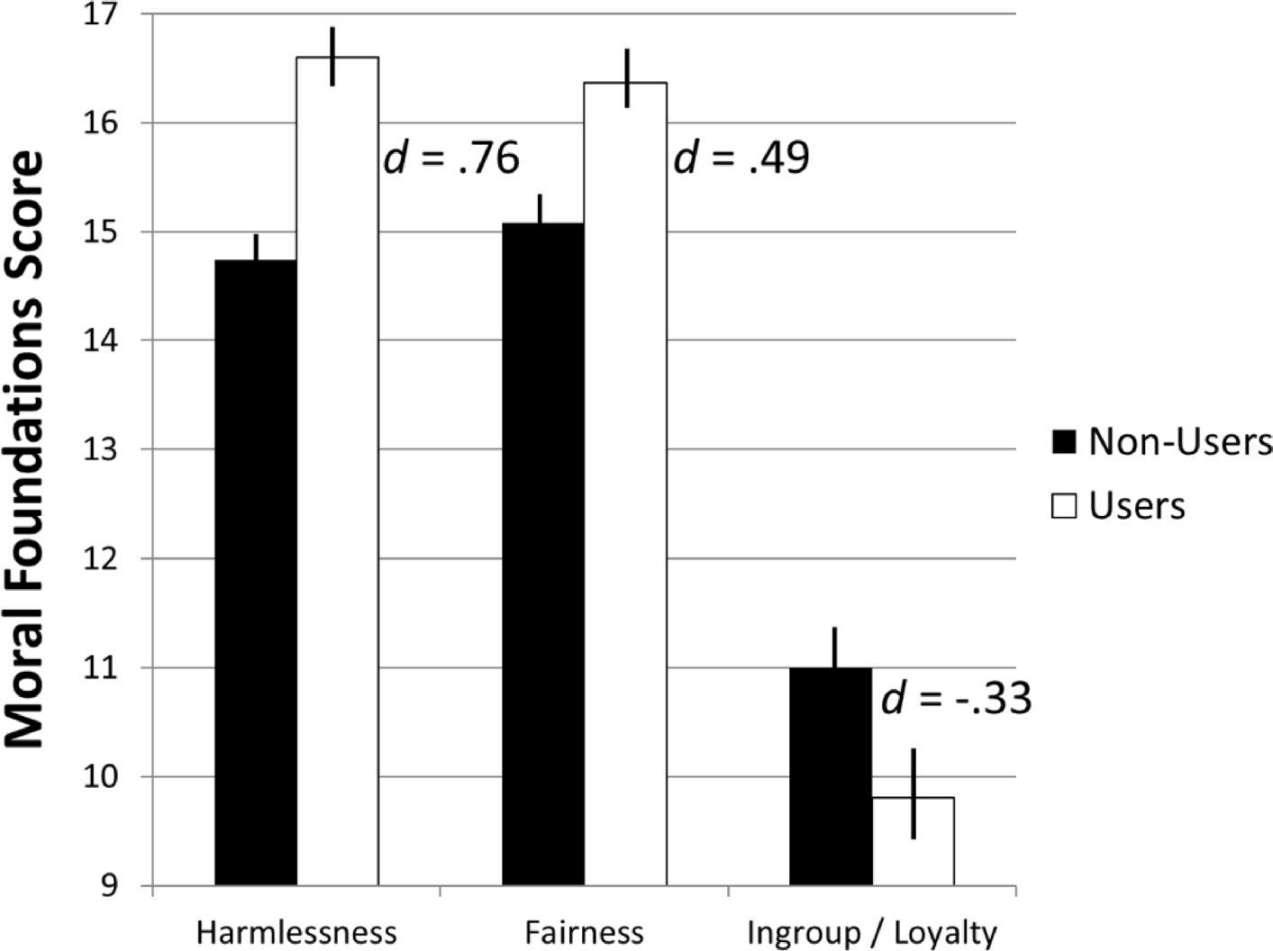
Group differences in moral foundation principles of harmlessness, fairness, and ingroup loyalty. Note. The harmlessness scores ranged from 6 to 20, fairness scores 8 to 20, and ingroup/loyalty scores 0 to 19.

Among the remaining variables, regressions excluding the interaction term and controlling for Sex, Age, Income, and Ethnicity showed significant main effects of User Group for: Prosocial Behaviors, *B* = 2.77, *t* = 2.05, *p* = .04; Empathy Quotient, *B* = 0.26, *t* = 2.48, *p* = .02; and the moral foundation principles associated with a sense of Harmlessness, *B* = 1.88, *t* = 4.68, *p* < .01; Fairness, *B* = 1.25, *t* = 2.84, *p* < .01; and Ingroup Loyalty, *B* = − 1.23, *t* = − 2.03, *p* = .04. As shown in Fig. 2a and b, cannabis users reported significantly higher levels of Prosocial Behaviors (*d* = .34) and Empathy (*d* = .36). Figure 3 shows users also scored higher on the moral foundations of Harmlessness (*d* = .76) and Fairness (*d* = .49), and scored lower on Ingroup Loyalty (*d* = − .33), as compared to non-users.

The partial correlations shown in Table 3 examine the associations between recency of cannabis usage and the Prosocial Behaviors, Empathy Quotient, and Moral Foundations associated with a sense of Harmlessness, Fairness, and Ingroup Loyalty, controlling for age, sex, income, and ethnicity among the entire sample. While the latency since last cannabis use was not associated with Prosocial Behaviors, recency of usage was significantly and linearly associated with higher scores on the Empathy, Moral Harmlessness, and Moral Fairness scores, and lower Moral Ingroup Loyalty scores (*p*s < .05). Partial correlations between the recency since the female participant’s last reported use of cannabis and their overall Aggression and Physical Aggression scores, controlling for age, income, and ethnicity, were not significant (*p*s > .10). Partial correlations between usage latency and the Agreeableness personality dimension among men showed a significant linear association (*r* = .36, *p* < .01).

**Table 3:** Partial correlations between latency since last cannabis use and prosocial behaviors, empathy quotient, and moral foundations of harmlessness, fairness, and ingroup loyalty.

	Recency of cannabis usage	Prosocial behaviors	Empathy quotient	Moral harmlessness	Moral fairness	Moral ingroup loyalty
Prosocial behaviors	.18*	–				
Empathy quotient	.27**	.50***	–			
Moral harmlessness	.38***	.35***	.31***	–		
Moral fairness	.27**	.35***	.31***	.60***	–	
Moral ingroup loyalty	− .24**	.14	.07	− .12	.12	–

*n* = 118, *p < .05, **p < .01, ***p < .001.

We found no overall group differences between cannabis and non-cannabis users for the Aggression sub-measures of Verbal, Anger, and Hostility, the Peer Trust measures (overall and for Predictability, Dependability and Faith), four of the Big 5 Personality measures, Moral Foundations scores overall and for sub-measures Respect for Authority and Purity, and reported Facial Threat Perceptions. Together with the positive association between cannabis use and Prosocial Behaviors, the Empathy Quotient, Moral Harmlessness, Moral Fairness, and Agreeableness, these null results suggest a positive effect from cannabis use on prosociality with no evidence of a countervailing increase in antisocial motivations in the overall sample.

## Discussion

The current study adds to the scientific literature by showing both sex-specific and more universal associations between cannabis use and several social psychological constructs associated with the concept of prosociality. Many of the constructs showed a linear relation with the recency of the last time the participants consumed cannabis, suggesting an immediate effect that diminishes over time. The general effect is consistent with a shift in perceptions that prioritize the role of prosocial behaviors, social empathy, benevolence, and fairness, independent of in-group identifications. In men, cannabis exposure was also associated with higher scores on the agreeableness personality dimension, matching levels of non-using females. The exception to this general shift towards heightened trait-levels of prosociality was the finding of higher aggression scores among female cannabis users, as compared to non-users. However, because the aggression scores were uncorrelated with recency of usage, they are likely the result of selection effects, whereby females that experiment with cannabis are more likely to score higher on trait aggression, on average, as compared to females that choose not to experiment with cannabis, rather than, for example, the possibility that cannabis directly increases aggressive behaviors in women^21^.

Our results are instead consistent with research showing the acute THC intoxication is usually associated with dampened aggression, and positively related to subjective feelings of openness, peace, joy, wonder, spirituality, and a heightened sense of connection to the universe^22,28,33,51,52^, with some researchers estimating that frequent cannabis use can increase an individual’s sociability by as much as 68.4%, thinking profoundly by 31.4%, happiness by 16.1%, feeling nice or pleasant by 20.9%, insight into others by 11.9%, and insight into oneself or personal growth by 8.7%^22^. Similar findings show cannabis users report a greater ability to empathize with others when shown discrete facial expressions of emotion, as compared to non-users^41^. Still, cannabis’ effects on aggressive behaviors are likely moderated by a host of genotypic (e.g., HTR2B variation^53^;) and circumstantial factors, including individual differences in baseline and diurnal mental and physical health states, recent and past experiences, social contextual and nonsocial environmental factors, and the natural heterogeneity of the *Cannabis* plant in and of itself.

The biopsychosocial mechanisms by which cannabis induces a transformation in perceptual functioning have not yet been the subject of full direct investigation, but can be interpreted in the context of broad behavioral and neurocognitive frameworks of affective behaviors and emotionality. From an ethological perspective, trustworthiness cues, or submissive affective displays are the types of behaviors that humans express in order to strengthen the intimacy and reliability of their relationships^3^. Cannabis usage may, therefore, induce the expression of communicative gestures, such as self-described and demonstrated empathy, in ways that effectively increase users’ social desirability and the overall reliability of their social spheres in ways that promote a state of psychosocial homeostasis^3^. The ability to appreciate another person’s suffering is an integral component of prosociality, and greatly increases the likelihood that people will engage in actual acts of beneficence^54–56^. Another core component of prosociality, moral identity, outlines the individual’s sense of rightfulness and wrongfulness, and therefore, encourages and promotes what the individual considers prosocial behaviors and discourages antisocial behaviors^57,58^. Many researchers believe that morality becomes ingrained during adolescence^59–63^, and further research is needed to better understand how cannabis exposure at different stages throughout development may affect short-term and/or long-term changes in social psychological functioning.

Pharmacologically, consumption of the cannabis plant evokes feelings of well-being via activation of the endocannabinoid system, which helps regulate stress responses and reward motivations^64^. One of the most commonly studied endocannabinoids is called anandamide, which means ‘supreme joy’ in classical languages^65^, and in clinical samples, aggressive tendencies are associated with low basal levels of this naturally occurring endocannabinoid, suggesting a link between endocannabinoid deficiencies and aggression^33^. Cannabis users have also been shown to produce reduced amygdala reactivity to threatening social stimuli^39^, and cannabis usage rapidly amplifies one’s sense of happiness, optimism, and well-being^66^, with large national databases showing the most frequently perceived side-effects of medical cannabis usage, recorded in real-time, are a heightened sense of feeling “relaxed”, “peaceful”, and “comfy”^28^. These findings are in direct contrast to findings from other intoxicants, such as alcohol, that are well-established to cause acute increases in hostility and aggression^26^.

Nonetheless, the current study is not without limitations. Our cross-sectional analyses do not enable us to track participants over time, in particular, pre- and post-cannabis use, preventing an analysis of the within-user effects of cannabis on our social construct measures. Likewise, the convenience sample of university, undergraduate, psychology students included is fairly small (146 individuals) and may not be representative of the wider community, thus limiting the generalizability of our findings. For example, college students often differ from non-students in basic demographic characteristics (e.g., age, SES, health status), as well as how individuals encounter and are informed about the usage and effects of cannabis. It is possible, for example, that non-college students may have cannabis-related experiences coupled with harsher life circumstances and less positive events, rendering differential effects of cannabis use based on psychologically-conditioned responses. However, the university from which the current sample was recruited is designated as a “majority-minority” institution, with ethnic and racial compositions similar to the wider region, and includes a large proportion of “non-traditional” students (e.g., lower SES than national averages). Thus, the study’s limitations should be weighed against its unique strengths of being able to contrast (pre-COVID) normative psychological functioning among a relatively homogenous sample of healthy, young adults.

In conclusion, this is among the first studies to demonstrate a direct association between non-medical cannabis use and positive psychological outcomes in otherwise healthy young adults. Studies have shown that people develop their personalities during adolescence and young adulthood^1,67–69^, when prosocial behaviors and habits are also formed^70,71^, rendering the need for further basic psychological research in these age groups, especially among cannabis consumers outside of our sample, such as those with physical or mental health disorders. For example, if regular consumption of the *Cannabis* plant promotes prosociality, this raises the possibility of its use as an adjunctive therapy, e.g., among people being treated for ‘conduct disorders,’ such as the majority of incarcerated individuals in the U.S. Or, perhaps, cannabis may simply prove useful among people seeking to incorporate a heightened sense of prosociality in their daily activities and psychological perspectives. Of course, the benefits of any such use should be carefully weighed against potential costs to health and financial wellbeing.

## Supplementary Information

Supplementary Information.
