# Supplementary material for: Cannabis consumption and prosociality
Source: Sci Rep. 2022 May 19;12:8352. doi: 10.1038/s41598-022-12202-8 (PMC9119984; doi:10.1038/s41598-022-12202-8)
Supplement: Supplementary file 1 — Supplementary Information. [file 41598_2022_12202_MOESM1_ESM.docx]

**Supplemental Materials**

Descriptions of surveys.

*Aggression Questionnaire* (Buss & Perry, 1992). 12 items, 4 subscales (physical aggression, verbal aggression, anger, and hostility), scored 1 to 5 (from *extremely uncharacteristic of m*e to *extremely characteristic of me*). Example items: “Some of my friends think I am a hothead.” “If I have to resort to violence to protect my rights, I will.” “When people are especially nice to me, I wonder what they want.” “I tell my friends openly when I disagree with them.”

*Trust Scale* (Rempel, Holmes & Zanna, 1985). 18 items, 3 subscales (predictability, dependability, and faith in others), scored -3 to 3 (from *strongly disagree* to *strongly agree*). Example items: “I know how my peers are going to act.” “My peers can always be counted on to act as I expect.” “I have found that my peers are thoroughly dependable people, especially when it comes to things that are important.”

*Prosocial Behaviors* *Scale* (Caprara, Steca, Zelli, Capanna; 2005). 16 items, scored -2 to 2 (from *never* to *always*). Example items: “I try to help others.” “I spend time with those friends who feel lonely.”

*Empathy Quotient* (Baron Cohen & Wheelwright, 2004). 22 items, scored -3 to 3 (from *strongly disagree* to *strongly agree*). Example items: “I really enjoy caring for other people.” “I am good at predicting what someone will do.”

*Ten-Item Personality Inventory* (Gosling, Rentfrow, & Swann, 2003). 10 items, 5 subscales (extraversion, agreeableness, conscientiousness, emotional stability, and openness to new experiences), scored -3 to 3 (from *disagree strongly* to *agree strongly*). Example items: “Rate the extent to which the pair of traits applies to you?” “Extraverted/enthusiastic.” “Critical/quarrelsome.” “Dependable/self-disciplined.” “Anxious/easily upset.” “Open to new experiences/complex.”

*Moral Foundations Questionnaire* (Graham et al., 2011). 20 items, 2 parts, 5 subscales (harm/care, fairness/reciprocity, ingroup loyalty, authority/respect, purity/sanctity). Scored 0 to 5 (from *not at all relevant* to *extremely relevant* [part 1] and from *strongly disagree* to *strongly agree* [part 2]). Example items (part 1): “To what extent are the following considerations relevant when deciding right and wrong (part 1):” “Whether or not someone suffered emotionally.” “Whether or not some people were treated differently than others.” “Whether or not someone’s action showed love for his or her country.” “Whether or not someone showed a lack of respect for authority.” “Whether or not someone violated standards of purity and decency.” Example items (part 2): “Compassion for those who are suffering is the most crucial virtue.” “When the government makes laws, the number one principle should be ensuring that everyone is treated fairly.” “I am proud of my country’s history.” “Respect for authority is something all children need to learn.” “People should not do things that are disgusting, even if no one is harmed.”

*Facial threat perceptions* (created with FaceGen software: Modeler 2.0, Singular Inversion Inc.). 6 facial stimuli with different combinations of expressions, 4 possible participant responses of the most representative emotion depicted (*sadness*, *joy*, *fear*, and *anger*) coded as affiliative (joy or sadness coded 0) or avoidant (anger or fear coded 1). Example: **
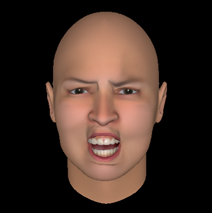
**
